# Impact of single-nucleotide variants and individual characteristics on adverse events of L-asparaginase in children with acute lymphoblastic leukemia

**DOI:** 10.3389/fphar.2024.1423049

**Published:** 2024-10-24

**Authors:** Jesús Alonso Gándara-Mireles, Ismael Lares-Asseff, Elio Aarón Reyes Espinoza, Verónica Loera Castañeda, Lourdes Patricia Córdova Hurtado, Flor de María Reyes Gutiérrez, Antonio Sandoval-Cabrera, Ignacio Villanueva Fierro, Julio Cesar Grijalva Ávila, Claudia Castro Arreola, Leslie Patrón-Romero, Horacio Almanza Reyes

**Affiliations:** ^1^ Department of Genomics, Interdisciplinary Research Center for Regional Comprehensive Development Durango Unit, National Polytechnic Institute (IPN), Durango, Mexico; ^2^ Latin American Network for the Implementation and Validation of Pharmacogenomic Clinical Guidelines (RELIVAF), Santiago, Chile; ^3^ Pediatric Hemato-Oncology Service, State Cancer Center (CECAN), Durango, Mexico; ^4^ Maternal and Child Institute of the State of Mexico (IMIEM), Toluca, Mexico; ^5^ Faculty of Medicine of the Autonomous University of the State of Mexico, Toluca, Mexico; ^6^ Faculty of Medicine and Psychology, Autonomous University of Baja California, Tijuana, Mexico

**Keywords:** single-nucleotide variants, L-asparaginase, adverse events, leukemia, children

## Abstract

**Introduction:**

L-Asparaginase (L-Asp) is a key drug in the treatment of acute lymphoblastic leukemia (ALL); however, it is commonly associated with the occurrence of adverse events (AE). Risk factors such as age, sex, nutritional status, and some single nucleotide variants (SNVs) in specific genes could be related to hypersensitivity reactions to L-Asp. The objective of this study was to identify the influence of individual characteristics and three SNVs in the *GRIA1* and *NFATC2* genes on the occurrence of the most significant adverse events caused by the use of L-Asp in Mexican children with ALL.

**Methods:**

Eighty-five children from ages 0–17 years old diagnosed with ALL were included. The patients were treated at two hospital centers in Mexico. The SNV genotypes of the *GRI1A* and *NFATC2* genes studied were examined using real-time qPCR. The evaluation of AE was carried out according to the Common Terminology Criteria for adverse events, and the determination of anti-L-Asp antibodies was conducted using Western blot immunoassay.

**Results:**

Homozygosity (AA) of the *GRIA1* rs4958351 SNV was significantly associated with the occurrence of AE with the use of L-Asp (OR = 4.05; 95% CI = 1.06 to 15.40, *p* = 0.04) and was strongly associated with the development of anti-L-Asp antibodies (OR = 3.4375, 95% CI = 1.04 to 11.25, *p* = 0.04). With this, we found a significant risk association for the SNV rs4958351 of the *GRIA1* gene. On the other hand, we did not find significant risk associations for the *GRIA1* rs6889909 and *NFATC2* rs6021191 SNVs, although other populations have shown a significant risk.

**Discussion:**

Our study has some limitations, such as the small sample size, the heterogeneity in adverse events due to the patients’ different regions of origin, and the limited ability to conduct a more detailed follow-up on pancreatitis. Additionally, since no significant associations were found between the *NFATC2* rs6021191 and *GRIA1* rs6889909 SNVs and the development of adverse events or the presence of antibodies due to the use of L-Asp, it is necessary to investigate new specific SNVs that may improve the efficacy and safety of treatment in Mexican children with ALL.

## Introduction

Acute lymphoblastic leukemia (ALL) is the most common type of leukemia in children and represents the leading cause of cancer in the pediatric population. The incidence can vary between regions and countries, but worldwide, it is estimated that approximately 1–4 per 100,000 children will develop ALL each year (Temple et al., 2023; Burkhardt and Hermiston, 2019). In Mexico, according to the National Institute of Public Health, childhood cancer is the leading cause of death in children between the ages of 5 and 14. Among the different types of cancer, ALL is the most prevalent, accounting for 50% of childhood cancer (Infantil, 2021). Pediatric treatment of ALL with combination chemotherapy regimens is essential in order to achieve remission in patients (Fonseca et al., 2021). One of the key drugs in these treatment protocols is L-asparaginase (L-Asp), an agent that has been in use for more than 30 years; it was the first enzyme used in clinical practice as an antineoplastic agent (Kumar et al., 2014). The therapeutic mechanism of action of L-Asp is to reduce the circulating concentration of the amino acid asparagine (Asp), thus leaving tumor cells without the basic nutrients of this amino acid for protein synthesis and causing their death (Pokrovskaya et al., 2022; Rajić et al., 2015). While L-Asp is a cornerstone in the treatment of ALL and is key to achieving remission, chemotherapy protocols that include L-Asp are commonly associated with the development of adverse events (AE). The most common adverse events are those related to hypersensitivity reactions, such as rash, anaphylactic shock, pain, swelling, and, in some cases, pancreatitis, which is an AE not related to hypersensitivity reactions (Thu Huynh and Bergeron, 2017; Gibson et al., 2021; Tosta et al., 2023).

The risk of AEs due to L-Asp has not been elucidated in the pediatric setting; however, it is modified by individual factors, including immunophenotype, chromosomal abnormalities, SNVs, younger age, sex, nutritional status, and the potential development of anti-L-Asp antibodies in each patient. Because L-Asp is an enzyme of non-human origin, the body sometimes recognizes it as a foreign agent and triggers an immune response that can cause AEs (Ramya et al., 2012). In addition, this immunological response by L-Asp causes a rapid plasma clearance of the drug, reducing its efficacy and requiring the need for multiple administrations and, thus, high rates of allergic reactions that become a cycle of events (Zhou et al., 2023). Cairo MS noted that AEs in children with ALL who were administered L-Asp as part of their induction protocol may represent a factor influencing the possibility of early termination of the therapy, and for this reason, he proposed an analysis for future modifications of L-Asp treatment (Cairo, 1982). In the same way, Schmidt et al. found that some variables, such as age over 10 years and female, had an association with the occurrence of AEs due to L-Asp (Schmidt et al., 2021). On the other hand, some contributions of SNVs associated with the risk of AEs by L-Asp have been scarcely studied; however, because L-Asp induces an immune response, some genes may have an influence on this response.

As a result of their studies, Rajić et al. and Ali et al. reported that some SNVs could be related to L-Asp hypersensitivity, specifically variants in the glutamate ionotropic receptor AMPA-type subunit 1 (*GRIA1*) and the nuclear factor of activated T cells cytoplasmic, calcineurin-dependent 2 (*NFATC2*) (Rajić et al., 2015; Ali et al., 2023). As a result of their studies in native Slovenian patients, they demonstrated a strong risk factor for L-Asp hypersensitivity in the presence of the intronic SNV rs4958351 of the *GRIA1* gene. This gene is located on the long arm of chromosome 5, region 3, band 3, sub-band 2 (5q33.2); within this cytogenetic locus, there is a group of genes coding for cytokines and other genes related to the immune system (Rajić et al., 2015). In the same sense, Fernández et al. used a genome-wide approach to identify loci associated with L-Asp hypersensitivity in children with ALL enrolled in the St. Jude Children’s Research Hospital protocol and reported that an intronic SNV rs6021191 in the nuclear factor of activated T cells 2 (*NFATC2*) had a strong association with L-Asp hypersensitivity (Fernandez et al., 2015).

Given the importance and frequency of the occurrence of AEs due to the use of L-Asp in patients with ALL and the fact that there are no studies of this type in the Mexican population, our study aims to identify individual patient characteristics and risk associations of the SNVs *GRIA1* rs4958351, rs6889909, and *NFATC2* rs60211 on the development of L-Asp-related adverse events, such as hypersensitivity reactions and pancreatitis, in children with ALL undergoing treatment.

## Materials and methods

This is a case–control, prospective, descriptive, longitudinal, and comparative study. Eighty-five children with ALL who were treated with L-Asp were included, of whom 19 experienced some type of AEs related to hypersensitivity reactions according to the Common Terminology Criteria for Adverse Events (v4.03, National Cancer Institute, 2013) (National Cancer Institute, 2009), or non-hypersensitivity reactions such as pancreatitis. Sixty-six did not experience any type of AE. Patients were divided into two groups according to whether they experienced (cases) or did not experience (controls) AEs from the use of L-Asp. All of them were treated at two different hospital centers in Mexico; 55 were from the Pediatric Haemato-Oncology service at the SSA Cancerology State Center (CECAN) in Durango, México, and 30 from the Maternal and Child Institute of the State of Mexico, Mexico. All patients were diagnosed with ALL according to the Franco-American-British Association of Hematology (FAB) criteria (Bennett et al., 1976). This investigation was approved by the Ethics Committee and Research Ethics Committee of the General Hospital of Durango, Mexico, with registration number 593/022, by the Research Ethics Committees of CECAN, SSA of Durango, Mexico with registration number 005, and by the Research and Ethics Committee of the Center for Research and Advanced Studies in Health Sciences of the School of Medicine, U.A.E.M., State of Mexico, Mexico, in accordance with the Declaration of Helsinki and the General Health Law of Mexico.

### Hypersensitivity evaluation

Clinical symptoms of hypersensitivity reactions grade 1 to 5 were considered as defined by the Common Terminology Criteria for Adverse Events (v4.03 National Cancer Institute 2013) that included skin rash, erythema, urticaria, fever ≥38°C, symptomatic bronchospasm, and anaphylactic shock (National Cancer Institute, 2009).

### Evaluation of pancreatitis associated with L-asparaginase

In order to establish the diagnosis of pancreatitis due to L-Asp, all patients were followed up for amylase and lipase enzyme determination even when the patients were asymptomatic. In the presence of clinical symptoms and elevated levels of these enzymes, a computed tomography (CT) scan (Al-Khazraji et al., 2017) was performed to confirm the diagnosis of pancreatitis (Wolthers et al., 2017).

### Genotyping

DNA was obtained from whole blood using the “DTAB CTAB” DNA extraction procedure (Gustincich et al., 1991). Its integrity and purity were determined by horizontal electrophoresis in a 1% agarose gel, followed by staining with Red Texas, and it was quantified by spectrophotometry using a NanoDrop (Thermo Scientific, Pleasanton, California, United States). The specific SNV of each gene was determined by real-time qPCR using TaqMan in StepOne thermocycler (Applied Biosystems, Pleasanton, California, United States) with specific probes for each SNV (Thermo Scientific, Pleasanton, California, United States); GRIA1 rs4958351 (C__29349605_10), GRIA1 rs6889909 (C__29201674_10), and NFATC2 rs6021191 (C__29689803_10).

### Detection of anti-L-Asp antibodies

For the specific detection of anti-L-Asp antibodies, a Western blot immunoassay was conducted using a primary antibody specific for human IgE from Santa Cruz Biotechnology (Lot #DDX0290P-100, 0.5 mg/mL) and a secondary specific antibody HRP-conjugated for IgE (Lot no. B0422, 40 ug/0.1 mL) from Santa Cruz Biotechnology. A primary antibody for monoclonal IgG from Mouse-IgG (lot n.º C1122, 200 ug/mL) was sourced from Santa Cruz Biotechnology. An imaging system (ChemiDoc from BIO-RAD) was used to read the samples after completion of the assay.

The Western blot method was conducted using serum samples from cases and controls. These samples were previously subjected to polyacrylamide gel electrophoresis (SDS-PAGE) to separate the proteins according to their molecular weight. The separated proteins were then transferred to a nitrocellulose membrane and incubated with a specific primary antibody. In the case of IgE, this primary antibody bound to any IgE present in the sample, and then the membrane was incubated with an enzyme-conjugated secondary antibody that recognized the primary antibody and allowed detection of the specific protein bands. Additional controls were performed to verify the specificity of anti-L-Asp antibody detection. These controls included performing blocking assays, where samples were incubated with purified L-Asp prior to detection by Western blot to assess the ability of L-Asp to block the detection signal.

### Evaluation of body mass index for age percentile growth charts for boys and girls

The body mass index for age percentile growth charts for boys and girls published by the Centers for Disease Control and Prevention (CDC) were used to categorize patients. Underweight was defined as weight below the fifth percentile, normal weight as above the fifth percentile to less than the 85th percentile, overweight between the 85th and 94th percentile, and obesity was considered equal to or greater than the 95th percentile (Centers for Disease Control and Prevention, 2021).

### Statistical analysis

Data are presented as mean ± SD or proportions. Differences between numerical variables were determined using Student's t-test for independent samples and the χ2 test (Fisher’s exact test) for categorical variables. Multivariate analysis was performed with a correspondence test using TIBCO Statistica 13.3 software (Weiss, 2008; Electronic Statistics Textbook, 2023). Genotypic frequencies were obtained by direct counting, and the Hardy–Weinberg equilibrium (HWE) was calculated using χ2 and goodness-of-fit statistics. Both analyses were carried out using the SNPstats package (http://bioinfo.iconcologia.net/SNPstats) (Solé et al., 2006). The association between SNV and AEs by L-Asp use was evaluated with multivariate logistic regression analysis. The risk association analysis between the studied SNV and the presence of anti-L-Asp antibodies was evaluated with the odds ratio (OR) test (McHugh, 2009). The statistical significance was set at *p* < 0.05 with a 95% confidence interval (CI). PASW Statistics 18.0 software was used for statistical analysis.

## Results

The demographics of children with ALL with AEs (cases) and without AEs (controls) with the use of L-Asp are shown in Table 1, showing statistically significant differences between children according to their body mass index (BMI) (*p* = 0.04).

**TABLE 1:** Comparison between the demographic aspects of patients with ALL under treatment with L-Asp grouped by cases and controls.

Variables	Cases n = 19	Controls n = 66	p**
Age (years)	9.65 ± 2.4*	10.69 ± 2.6*	0.06
Sex (M/F)	11/8	35/31	0.24
Weight (kg)	36.8 ± 11.8*	32.7 ± 9.11*	0.10
Height (cm)	136.3 ± 22.2*	134.1 ± 20.01*	0.22
BMI	19.1 ± 2.5	17.3 ± 3.3	0.042

F = female; M = male; kg: kilogram; cm: centimeter; BMI: body mass index; *SD: standard deviation; *p*: Statistical significance was established at *p* < 0.05; **Student’s t-test; Groups by age n = 85.

Table 2 shows the description of the AEs presented in patients grouped by age, with the highest percentage of AEs in children aged 7–12 years; anaphylactic shock and pancreatitis were the most common adverse events in patients (26.3% each). No statistically significant differences were found between the different groups (*p* = 0.1262). Table 3 shows the genotypic and allelic frequencies of the SNVs studied. No statistically significant association was found between cases and controls for any of the SNVs. All SNVs studied for our groups were in Hardy–Weinberg equilibrium (HWE).

**TABLE 2:** Description of the adverse effects presented in the patients included in the study grouped by age.

Type of adverse effect	1–6 years (n = 6)	7–12 years (n = 10)	13–18 years (n = 3)	%	p*
Pancreatitis	1	3	1	26.3	
Localized pain	1	3	0	21.1	
Fever	0	3	0	15.8	0.1262
Rash	0	1	1	10.5	
Anaphylactic shock	4	0	1	26.3	
%	31.6	52.6	15.8		

n = 19 participants included; %: Representative fraction considering the total number of patients as 100%; *p*: Statistical significance was established at *p* < 0.05; * chi-squared test = 12.6033.

**TABLE 3:** Genotypic and allelic frequencies of the SNVs studied grouped by cases and controls.

Allele	Genotype	Cases n = 19	n (%)	Controls n = 66	n (%)	p*	HWE
*GRIA1* rs4958351	WT (GG)	5	26.3%	27	40.1%	—	—
	HT (GA)	6	31.6%	24	36.4%	0.2256	YES
	MH(AA)	8	42.1%	15	22.7%	—	—
	Alleles						
	Major allele (G)	16	42.1%	78	59.1%	0.0643	—
	Minor allele (A)	22	57.9%	54	49.9%	—	—
*GRIA1* rs6889909	WT (CC)	6	31.6%	30	45.4%	—	—
	HT (CG)	7	36.8%	20	30.2%	0.5554	YES
	MH(GG)	6	31.6%	16	24.2%	—	—
	Alleles						
	Major allele (C)	19	50%	80	60.7%	0.2441	—
	Minor allele (G)	19	50%	52	39.3%	—	—
*NFATC2* rs6021191	WT (AA)	10	52.6%	28	42.4%	—	—
	HT (AT)	7	36.8%	19	28.8%	0.2661	YES
	MH(TT)	2	10.6%	19	28.8%	—	—
	Alleles						
	Major allele (A)	27	71%	75	56.8%	0.1156	—
	Minor allele (T)	11	29%	57	43.2%	—	—

n = 85 participants included; *p*: Statistical significance was established at *p* < 0.05; *Fisher’s exact test; HWE: Hardy–Weinberg equilibrium; WT: wild type; HT: heterozygous; MH: mutated homozygote.

The crude analysis of the allelic state through inheritance models (Table 4) showed that the *GRIA1* rs4958351 variant was significantly associated with the risk of AE occurrence in the codominant model in the mutated genotype (AA) (OR = 4.05; 95% CI = 1.06 to 15.40, *p* = 0.04); the mere presence of a mutated allele (A) confers a statistically significant risk of AEs with the use of L-Asp (OR = 2.47; 95% CI = 1.17 to 5.21, *p* = 0.01). In addition, the recessive model shows that two copies of the mutated allele (AA homozygosity) have an increased risk of AEs due to the use of L-Asp in children with ALL under treatment (OR = 3.06; 95% CI = 1.05 to 8.91, *p* = 0.04). We found no significant associations for the *GRIA1* rs6889909 and *NFATC2* rs6021191 SNVs in our population.

**TABLE 4:** Association by inheritance modeling of *GRIA1* rs4958351, rs6889909, and *NFATC2* rs6021191 genetic variants with adverse events from the use of L-Asp.

Allele	Codominant model	Yes (n = 19)	No (n = 66)	OR	IC	p*
*GRIA1* rs4958351	WT (GG)	4	27	1	—	—
	HT (GA)	6	24	1.6875	0.4248 to 6.7043	0.4572
	MH(AA)	9	15	4.0500	1.0644 to 15.4096	0.0402
	Major allele (G)	14	78	0.5192	0.2571 to 1.0485	0.0676
	Minor allele (A)	24	54	—	—	—
	Minor allele (A)	24	54	2.4762	1.1757 to 5.2154	0.0170
	Major allele (G)	14	78	—	—	—
	Dominant model				0.1152 to 1.2881	
	WT (GG)	4	27	0.3852		0.1214
	HT (GA) +MH(AA)	15	39	—	—	—
	Recessive model			—	—	—
	WT (GG) + HT (GA)	10	51			
	MH (AA)	9	15	3.0600	1.0508 to 8.9108	0.0403
*GRIA1* rs6889909	Codominant model					
	WT (CC)	06	30	1	—	—
	HT (CG)	07	20	1.7500	0.5123 to 5.9781	0.3719
	MH(GG)	06	16	1.8750	0.5192 to 6.7708	0.3373
	Major allele (C)	19	80	0.6500	—	—
	Minor allele (G)	19	62	—	—	—
	Minor allele (G)	19	52	1.5385	0.7448 to 3.1779	0.2445
	Major allele (C)	19	80	—	—	—
	Dominant model				0.1877 to 1.6340	
	WT (CC)	06	30	0.5538		0.2844
	HT (CG) + MH (GG)	13	36	—	—	—
	Recessive model			—	—	—
	WT (CC) + HT (CG)	13	50			
	MH (GG)	06	16	1.4423	0.4710 to 4.4171	0.5213
*NFATC2 rs6021191*	Codominant model					
	WT (AA)	10	28	1	—	—
	HT (AT)	07	19	1.0316	0.3339 to 3.1870	0.9569
	MH (TT)	02	19	0.2947	0.0580 to 1.4985	0.1409
	Major allele (A)	27	75	1.8655	0.8542 to 4.0740	0.1177
	Minor allele (T)	11	57	—	—	—
	Minor allele (T)	11	57	0.5361	0.2455 to 1.1707	0.1177
	Major allele (A)	27	75	—	—	—
	Dominant model				0.5414 to 4.2000	
	WT (AA)	10	20	1.5079		0.4319
	HT (AT) + MH (TT)	09	38	—	—	—
	Recessive model			—	—	—
	WT (AA) + HT (AT)	17	47			
	MH (TT)	02	19	0.2910	0.0612 to 1.3836	0.1207

n = 85 participants included; *OR: odds ratio result; IC: 95% confidence interval; *p*: Statistical significance was established at *p* < 0.05. WT: wild type; HT: heterozygous; MH: mutated homozygote.

Table 5 shows the immunophenotypic characteristics and chromosomal abnormalities of the patients included in the study. Of the 19 patients with AEs, five had a T-ALL immunophenotype, and 14 had a B-ALL immunophenotype; in addition, two had t (9; 22) chromosomal abnormalities, and 11 had t (12; 21) chromosomal abnormalities. Of the five patients with pancreatitis, four had a T-ALL immunophenotype, and one had a B-ALL immunophenotype; in addition, two had t (9; 22) chromosomal abnormalities, and two had t (12; 21) chromosomal abnormalities. Of the patients who had antibodies and also had AEs associated with hypersensitivity reactions (14), one had a T-ALL immunophenotype, 13 had a B-ALL immunophenotype, none of them had t (9; 22) chromosomal abnormalities, and nine had t (12; 21) chromosomal abnormalities.

**TABLE 5:** Immunophenotypic characteristics and chromosomal abnormalities of the patients included in the study.

Category	Total patients	T-ALL immunophenotype	B-ALL immunophenotype	Chromosomal abnormalities t (9; 22)	Chromosomal abnormalities t (12; 21)
Total number of patients	85	5	80	2	11
Patients with adverse events	19	5	14	2	11
Patients without adverse events	66	0	66	0	0
Patients with pancreatitis	5	4	1	2	2
Patients with hypersensitivity	14	1	13	0	9
Patients with positive antibodies	17	1	13	0	9

**FIGURE 1:**
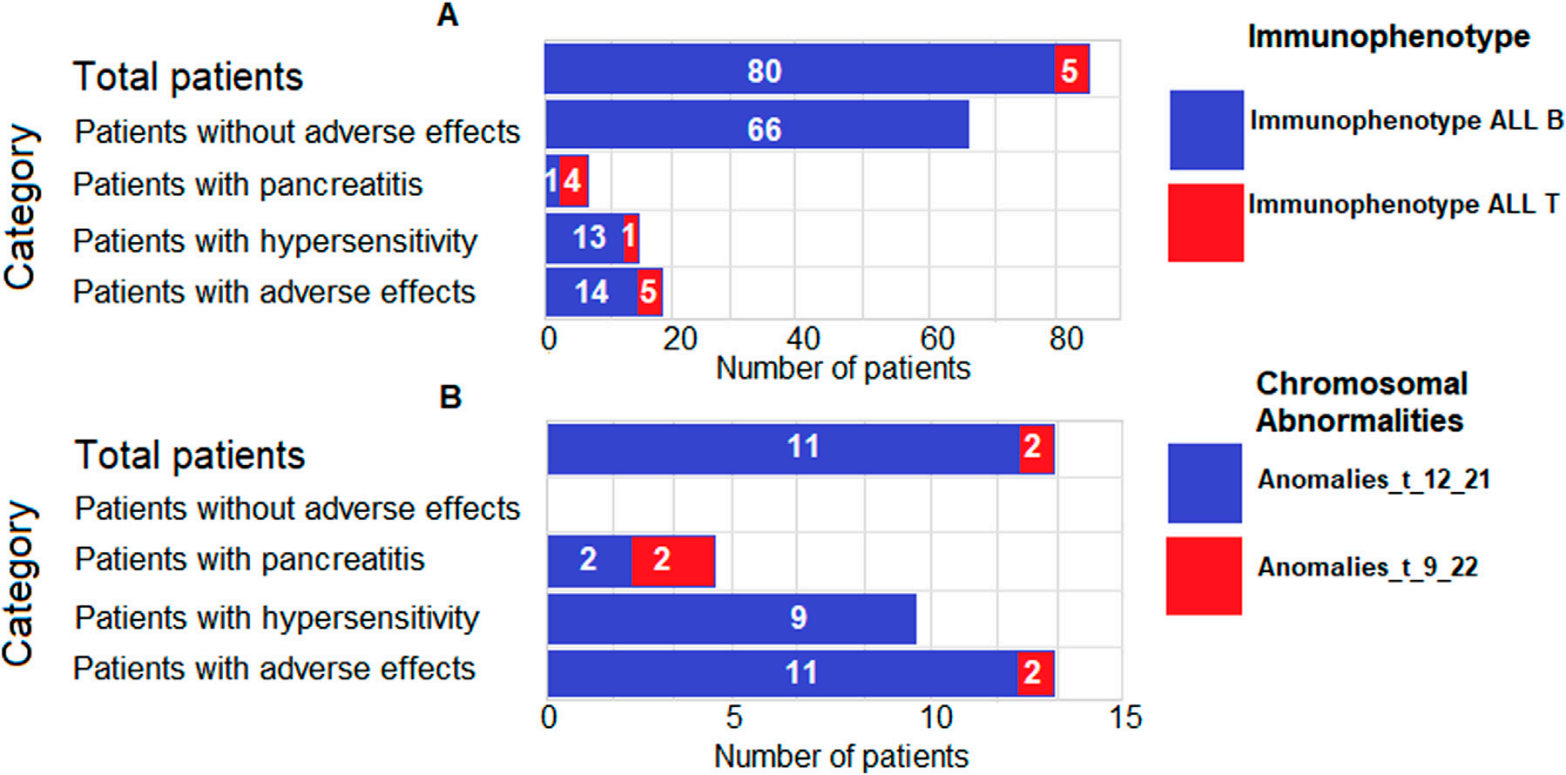
Immunophenotypic characteristics and chromosomal abnormalities of the patients included in the study. The bar chart illustrates the distribution of immunophenotypes (T-ALL and B-ALL) **(A)** and chromosomal abnormalities (t (9; 22) and t (12; 21)) **(B)** among different categories of patients with acute lymphoblastic leukemia (ALL). The bars represent the number of patients in each category, and the labels indicate the frequency of patients with each characteristic.

The immunophenotypic characteristics and chromosomal abnormalities of the patients included in the study are shown in Figure 1. All five patients with a T-ALL immunophenotype had AEs; four had pancreatitis, and one had hypersensitivity reactions. Furthermore, of the 13 patients with chromosomal abnormalities, two had t (9; 22), and both of those two patients developed pancreatitis.

Table 6 shows the association of the presence of antibodies with the different types of AEs, where five of the 19 patients (26.3%) presented pancreatitis, but none presented antibodies. On the other hand, 14 of 19 patients (73.7%) with AEs experienced hypersensitive reactions to L-Asp, including local pain, fever, rash, and anaphylactic shock. All of these patients tested positive for anti-L-Asp antibodies. Table 7 shows the associations of the genotypic status of the *GRIA1* rs4958351, rs6889909, and *NFATC2* rs6021191 SNV and the presence of antibodies by the odds ratio test in patients under treatment with L-Asp. We found a highly significant risk association between AA homozygosity in the *GRIA1* rs4958351 variant and the development of anti-L-Asp antibodies (OR = 3.43, 95%, CI = 1.04 to 11.25, *p* = 0.04). We found no significant associations for the *GRIA1* rs6889909 and *NFATC2* rs6021191 SNVs in our population.

**TABLE 6:** Association between the presence of antibodies and the different types of adverse events.

Category	Presence of antibodies	Absence of antibodies	Total
Patients with AE+			
Pancreatitis	0	5 (26.3%)	5 (26.3%)
Hypersensitivity	14 (73.7%)	0	14 (73.7%)
Subtotal AE+	14 (73.7%)	5 (26.3%)	19
Patients with AE−	3 (4.5%)	63 (95.5%)	66
Total	17	68	85

Groups n = 19; *: Chi-squared test = 19.0000; *p*: Statistical significance was established at *p* < 0.05.

**TABLE 7:** Associations of the genotype status of the *GRIA1* rs4958351, rs6889909, and *NFATC2* rs6021191 SNVs and the presence of antibodies by odds ratio testing in patients under treatment with L-Asp.

SNV	Genotype	Cases	Controls	OR	95% CI	p*
*GRIA1* rs4958351	WT (GG)	2	30	0.2278	—	0.0646
	HT (GA)+MH(AA)	12	41	—	0.0474 to 1.0941	—
	HT (GA)	5	25	1.0222	0.3089 to 3.3831	0.9713
	WT (GG)+MH(AA)	9	46	—	—	—
	MH(AA)	7	16	3.4375	1.0495 to 11.2587	0.0414
	WT (GG)+HT (GA)	7	55	—	—	—
*GRIA1* rs6889909	WT (CC)	4	32	0.4875	0.1396 to 1.7020	0.2600
	HT (CG)+MH(GG)	10	39	—	—	—
	HT (CG)	5	22	1.2374	0.3714 to 4.1222	0.7287
	WT (CC)+MH(GG)	9	49	—	—	—
	MH(GG)	5	17	1.7647	0.5202 to 5.9869	0.3621
	WT (CC)+HT (CG)	9	54	—	—	—
*NFATC2* rs6021191	WT (AA)	8	30	1.8222	0.5721 to 5.8043	0.3100
	HT (AT)+MH(TT)	6	41	—	—	—
	HT (AT)	5	21	1.3228	0.3959 to 4.4190	0.6495
	WT (AA)+MH(TT)	9	50	—	—	—
	MH(TT)	1	20	0.1962	0.0241 to 1.5997	0.1282
	WT (AA)+HT (AT)	13	51	—	—	—

SNV: single-nucleotide variants; *OR: odds ratio result; IC: 95% confidence interval; *p*: Statistical significance was established at *p* < 0.05. WT: wild type; HT: heterozygous; MH: mutated homozygote.

Table 8 shows the evaluation of the most important risk factors for the development of AEs that were found in our study. The T-ALL immunophenotype was significantly associated with the development of AEs (RR: 3.5, 95% CI: 1.2232–10.2828, *p* = 0.02). The chromosomal abnormality t (9; 22) was significantly associated with the development of AEs (RR: 4.0, 95% CI: 1.0937–15.3821, *p* = 0.04). On the other hand, BMI <10th percentile was significantly associated with the development of AEs (OR: 7.1, 95% CI: 1.3243–29.9384, *p* = 0.02).

**TABLE 8:** Risk factor evaluation.

Variable	Relative risk (RR)	95% CI	*p*-value
T-ALL immunophenotype	3.5	1.2232–10.2828	0.02*
Chromosomal abnormalities t (9; 22)	4.0	1.0937–15.3821	0.04*
Odds Ratio (OR)			
BMI <10^th^ percentile	7.1	1.3243–29.9384	0.02**

*Relative Risk. **Odds Ratio.

Table 9 shows a description of the nutritional status and the development of pancreatitis in patients with ALL treated with L-Asp. All patients who developed pancreatitis were below the 10th percentile in terms of their nutritional status, suggesting a possible association between poor nutritional status and the development of pancreatitis as an adverse event in patients treated with L-Asp. Furthermore, three of these patients were boys, and two were girls, highlighting the need to consider gender differences in the predisposition to develop pancreatitis in this context.

**TABLE 9:** Description of nutritional status by percentile for age and the development of pancreatitis in patients with ALL treated with L-Asp.

Patient number	Sex	Age at admission (years)	Percentile for age	Category BMI
1	F	8	<10	HW
2	F	10	<10	HW
3	M	5	<5	UW
4	M	14	<10	HW
5	M	7	5	HW

F = female; M = male; BMI: body mass index; HW: healthy weight; UW: underweight.

**FIGURE 2:**
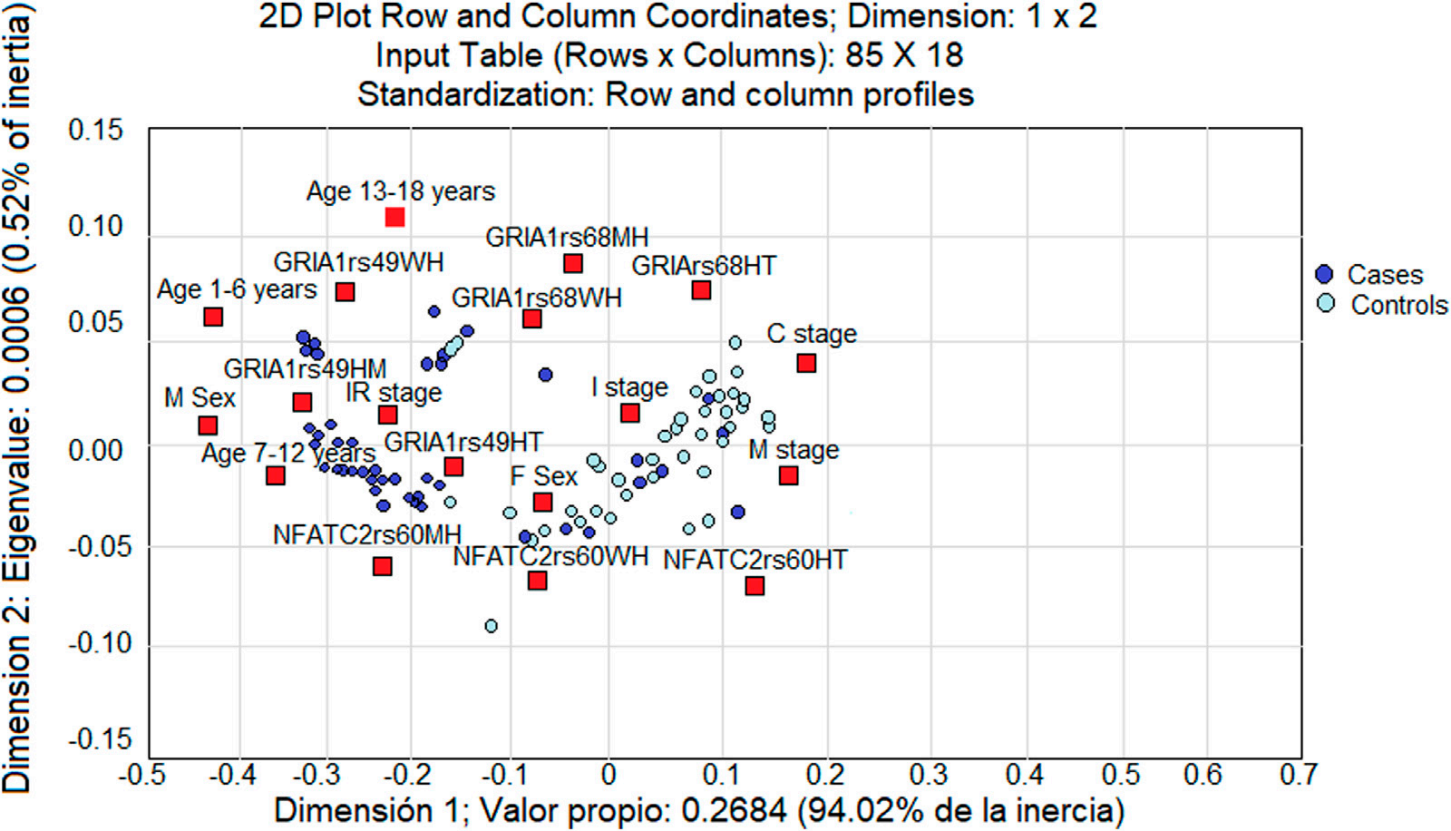
Multivariate analysis of the association between the variables of age, sex, treatment stage, and SNV studied (red squares) with all the patients involved in the study. The cases tend to be loaded on the left side of the graph, and controls appear on the right side (blue squares). The results are reflected as a value of “relative inertia” (the sum of the distances between the patients in the study and the variable studied).

A multivariate analysis was conducted to determine the importance of variables other than immunophenotype and chromosomal abnormalities (see Figure 2), showing the analysis by relative inertia between patients included in the study and variables of age, sex, stage of treatment, and SNV studied (red squares). The graph shows that the cases shift to the left side, whereas the controls shift to the right side (blue squares). In this multivariate analysis, there was a significant overall association between the variables and the patients studied (χ2 = 221.179, *p* = 0.0003). The highest associations between variables and patients who developed AEs are as follows: MH genotype of *GRIA1* rs4958351 SNV (relative inertia: 0.79944), age 7–12 years (relative inertia: 0.67984), and male sex (relative inertia: 0.65474).

## Discussion

L-Asp is a key drug in the treatment of pediatric patients with ALL. This enzyme-derived drug is essential to achieve remission; however, the occurrence of AEs has been reported during its use, mainly hypersensitivity reactions in up to 10%–70% of treated patients as well as pancreatitis in 2%–18% of the patients. The exact prevalence may vary depending on the treatment protocol used and the population studied (Hijiya and van der Sluis, 2016; Skipper et al., 2023). In this work, we studied a group of 85 Mexican patients with ALL from two hospital centers in the country who were under treatment with L-Asp, of whom 19 (22.4%) presented some type of AE, and 66 (77.6%) did not present any type of AE.

Previous studies suggest that nutritional status in cancer patients may influence treatment tolerance and response (Baracos, 2006; van der Meij et al., 2013). Orgel et al. reported a direct association between BMI and survival in children with leukemia receiving treatment (Orgel et al., 2016). In this regard, Orgel et al. found that a high BMI in children with ALL negatively impacts treatment tolerance and the ability to complete the treatment (Orgel et al., 2014). In our research, we found a statistically significant difference (*p* = 0.04) between our cases (patients with AE) and controls (patients without AE), with a higher BMI in patients who experienced AEs, which led us to believe that a higher BMI may influence the treatment’s response with L-Asp. We categorized patients according to the BMI-for-age percentile growth charts for boys and girls and found that patients who were below the 10th percentile had a statistically significant risk of developing pancreatitis (OR = 7.1438, 95% CI = 1.3243 to 29.9384, *p* = 0.02). This association between BMI and the response to a specific treatment, such as L-Asp, has been addressed in previous studies, and it highlights the importance of a nutritional status evaluation in pediatric cancer patients (van der Meij et al., 2013).

A higher incidence of AEs, especially anaphylactic shock and pancreatitis, was observed in children from 7 to 12 years of age. This is relevant to what was reported by Pui et al., who mentioned that the prevalence of leukemia is highest between the ages of 2 and 5 years; however, the older the age of children, the prevalence of AEs increases (Pui et al., 2008). Silva-Rodrigues et al. reported that adolescence is the age with the highest number of AEs due to chemotherapy (Silva-Rodrigues et al., 2022). Age is another factor that has been reported to be associated with AEs. Battistel et al. reported in their study on adverse events due to the use of L-Asp that male has a higher frequency of occurrence (Battistel et al., 2021); this coincides with what we found in our study because male, age, and the SNV rs4958351 of the *GRIA1* gene were among the variables with the highest association with the presence of AEs.

Several studies suggest that genetic variability may significantly influence the response to L-Asp in patients with ALL (Angiolillo et al., 2014). Rajić et al. reported some associations of genetic variants and AEs from the use of L-Asp in children from Slovenia with leukemia and solid tumors, specifically for the SNVs rs4958351, rs4958676, rs6889909, rs6890057, and rs10070447 of the *GRIA1* gene (Rajić et al., 2015). On the other hand, Fernandez et al. reported significant associations between the rs6021191 variant of the *NFATC2* gene and the development of AEs in patients with ALL who were being treated with L-Asp (Fernandez et al., 2015). In our work, through the analysis of inheritance models, we found a significant association between the SNV rs4958351 of the *GRIA1* gene and the occurrence of AEs. Specifically, in the codominant model in the mutated genotype (AA) (OR = 4.05; 95% CI = 1.06 to 15.40, *p* = 0.04), we found that the presence of one mutated allele (A) conferred a risk of AEs due to the use of L-Asp (OR = 2.47; 95% CI = 1.17 to 5.21, *p* = 0.01). In addition, the recessive model showed that two copies of the mutated allele (AA homozygosity) had an increased risk of AEs in children with ALL treated with L-Asp (OR = 3.06; 95% CI = 1.05 to 8.91, *p* = 0.04). Contrary to these studies, we did not find a significant association between SNVs rs6889909 of the *GRIA1* gene and rs6021191 *NFATC2* with the presence of AEs or the development of antibodies, which tells us about the variability that can occur between populations such as the Slovenian population and the Mexican population. In this sense, the Mexican population exhibits significant ethnic variability due to its rich history of mixing among Indigenous, European, and African populations over centuries. This ethnic diversity is reflected in the wide range of Indigenous groups present in the country, such as the Nahua, Maya, and Zapotec, among others, each with its own genetic identity (Silva-Zolezzi et al., 2009; Wang et al., 2008).

In this sense, the *GRIA1* gene (glutamate ionotropic receptor AMPA-type subunit 1) encodes a subunit of AMPA-type glutamate receptors, which are primarily involved in neurotransmission in the central nervous system (Sugimoto et al., 2010); however, some studies have suggested that glutamate receptors may have some influence in the immune response (Koda et al., 2023). A bidirectional communication between the nervous system and the immune system has been proposed. Neurotransmitters, such as glutamate, may affect immune cell function and inflammatory response (Pavlov and Tracey, 2012; Kipnis and Filiano, 2018). In our study, 14 of the 19 patients who had AEs experienced hypersensitivity reactions, and five had pancreatitis. All of the 14 patients with hypersensitivity reactions had anti-L-Asp antibodies, while none of the five patients with pancreatitis had anti-L-Asp antibodies. The risk association analysis by the odds ratio test between the SNV rs4958351, rs6889909 of the *GRIA1* gene, and rs6021191 of the *NFATC2* gene and the presence of antibodies showed a highly significant association between the AA homozygosity of SNV rs4958351 of the *GRIA1* gene and the development of anti-L-Asp antibodies, which is consistent with what has been previously reported in the literature for this variant and reinforces the relationship between genetics and the immune response to treatment.

Several clinical studies support the idea that the efficacy and safety of L-Asp treatment are closely related to the ALL subtype and specific genetic abnormalities, such as high-risk ALL subtypes, including BCR-ABL-positive ALL and T-ALL (Amylon et al., 1999). In addition, chromosomal abnormalities such as t (9; 22) and t (12; 21) may be associated with an unfavorable prognosis and reduced treatment effectiveness (Terwilliger and Abdul-Hay, 2017). In our study, we found that five of the 19 patients with AE had a T-ALL immunophenotype, and 14 of 19 had a B-ALL immunophenotype. Of these, two had the chromosomal abnormality t (9; 22) and 11 had the abnormality t (12; 21). Among the five patients with pancreatitis, four had a T-ALL immunophenotype, and one had a B-ALL immunophenotype. In addition, two of them had the chromosomal abnormality t (9; 22) and two had t (12; 21).

Our risk factor assessment analysis showed significant associations between T-ALL immunophenotype and t (9; 22) chromosomal abnormality with AEs, suggesting that these genetic abnormalities may be involved in the specific response to L-Asp in these patients and may be considered potential risk factors for treatment response.

### Limitations of the study

This study has several limitations that should be considered. First, as this is a longitudinal study, the observed associations do not allow for establishing direct causal relationships between genetic variants and L-Asp-induced AEs. In addition, the relatively small size of the sample, which consisted of children with ALL who were treated with L-Asp, reduces the generalizability of our results. Despite our best efforts to maximize the number of patients included, the limited availability of participants and budget constrained the scope of the study. However, we believe that the results obtained, although limited in terms of sample size, provide valuable insight into the genetic associations in this population and are consistent with those of previous studies that also used samples of similar size, such as Rajia et al. and Windsor et al., who included 76 and 60 patients, respectively (Rajić et al., 2009; Windsor et al., 2012). Future research with larger sample sizes will be needed to confirm the impact of genetic variants such as rs4958351 and rs6889909 of the *GRIA1* gene and rs6021191 of the *NFATC2* gene on the occurrence of AEs in this population. Another significant limitation is the heterogeneity of the AE results, which could be due to the fact that the patients came from two hospital centers located in different states of Mexico (Durango and Estado de México). The observed variability may be influenced by demographic and migratory differences between these regions. Importantly, due to sample size limitations and the low allele frequency of certain variants, we did not investigate rare missense variants in *GRIA1* and *NFATC2*. These variants typically have a lower allele frequency, which would have required a considerably larger sample size to obtain statistically significant associations. We acknowledge that this is a significant limitation and that future research should focus on the impact of these rare variants in larger and more diverse populations. Finally, the evaluation of L-Asp-associated pancreatitis was based on the different studies (determination of amylase and lipase enzymes) and a single computed tomography (CT) scan, which limited our ability to perform a more detailed follow-up of the clinical evolution of this complication. In future studies, incorporating more comprehensive diagnostic and follow-up methods would be beneficial for better characterizing this and other treatment-related complications.

### Strengths of the study

This study has several key strengths that reinforce the relevance and validity of our findings. First, this study is among the first multicenter investigations in Mexico to thoroughly assess the adverse events associated with L-Asp treatment in children with ALL. The multicenter nature of the study, which includes two hospital centers located in different states of the country (Durango and Estado de México), allows for greater diversity in the sample and, therefore, a better representation of the Mexican childhood population with ALL. The study used standardized methods for sampling, collecting, and analyzing data. This rigorous approach ensures the consistency and reproducibility of the results, minimizing potential biases and errors arising from different methodologies. Another key strength is the participation of a multidisciplinary committee composed of experts in oncology, molecular biology, clinical genetics, pharmacogenomics, pediatric hemato-oncologists, pediatricians, nurses, and other healthcare professionals. This diversity of experts allowed for a robust and comprehensive interpretation of the data, considering multiple perspectives and ensuring the scientific quality of the analysis. Finally, the study used a wide range of health indicators, including clinical assessments of hypersensitivity, diagnosis of pancreatitis, genotyping analysis, and nutritional status. This comprehensive approach not only provides a more complete understanding of the AEs associated with L-Asp but also allows for the identification of both genetic and clinical factors that may influence susceptibility to these AEs, providing significant clinical value. Furthermore, the selection of specific SNVs, such as *GRIA1* rs4958351 and *NFATC2* rs6021191, based on previous studies, reinforces the relevance of the variants studied in this population. While we recognize the limitations of sample size and genetic variability, we believe this approach provides a solid foundation for future larger studies that can validate and expand upon our findings.

## Future prospects

Our research highlights the importance of taking into account individual factors and genetic variations in the occurrence of AEs, such as hypersensitivity reactions and pancreatitis related to L-Asp, in children with ALL. These results emphasize the necessity for a thorough evaluation of individual and genetic traits to enhance treatment strategies and minimize risks. However, to expand and reinforce these observations, additional studies with larger and more diverse populations, including various ethnic groups, are essential. Furthermore, exploring other biomarkers that may affect and influence the response to L-Asp and conducting longitudinal studies to evaluate the long-term effects of these genetic variants on patient health would be beneficial. Incorporating advanced genomics and proteomics methods could also yield deeper insights into the mechanisms behind adverse events. These efforts will aid in personalizing treatments and improving the safety and efficacy of L-Asp therapy for pediatric patients.

## Conclusion

Our study shows statistically significant differences in BMI between children with ALL treated with L-Asp who experienced AEs and those without AEs. Furthermore, we found a significant association between the development of pancreatitis and BMI-for-age growth percentile in boys and girls below the 10th percentile, highlighting the importance of nutritional status in the occurrence of L-Asp-induced pancreatitis. The age group between 7 years and 12 years had the highest percentage of children with AEs, with anaphylactic shock and pancreatitis being the most common events (26.3% each). Multivariate analysis showed significant associations between age, sex, treatment stage, and the SNV studied in patients who developed AEs. It highlighted a significant relative inertia for men, associating them with the occurrence of AEs. Allelic status analysis by inheritance modeling revealed a significant association between the SNV rs4958351 of the *GRIA1* gene and the AE occurrence. The codominant and recessive models demonstrated increased risks, highlighting the importance of this SNV with the association of AE risk. The presence of antibodies was associated with hypersensitivity reactions, whereas none of the patients with pancreatitis developed antibodies, suggesting a possible immunologic pathway in hypersensitivity reactions and the importance of understanding the immune response in relation to AEs. A highly significant association was found between AA homozygosity of the SNV rs4958351 of the *GRIA1* gene and the development of anti-L-Asp antibodies, supporting the genotypic link in the immune response. In our population, we found no significant associations between the SNVs rs6889909 of the *GRIA1* gene and rs6021191 of the *NFATC2* gene. However, it is important to note that the Mexican population has great ethnic variability due to its rich history of mixing among Indigenous peoples, Europeans, and Africans. Finally, our risk factor assessment analyses showed significant associations between ALL-T immunophenotype and t (9; 22) chromosomal abnormality with AEs, suggesting that these genetic abnormalities may be involved in the specific response to L-Asp. These results may help in the effective and safe use of L-Asp in pediatric patients with ALL who are receiving treatment, but further study of new specific SNVs that have a significant impact on our population is needed.

## Data Availability

The original contributions presented in the study are included in the article/Supplementary Material, further inquiries can be directed to the corresponding author.
