# Supplementary material for: Impact of single-nucleotide variants and individual characteristics on adverse events of L-asparaginase in children with acute lymphoblastic leukemia
Source: Front Pharmacol. 2024 Oct 24;15:1423049. doi: 10.3389/fphar.2024.1423049 (PMC11540776; doi:10.3389/fphar.2024.1423049)
Supplement: Supplementary file 1 [file Presentation1.PPTX]

## Slide 1
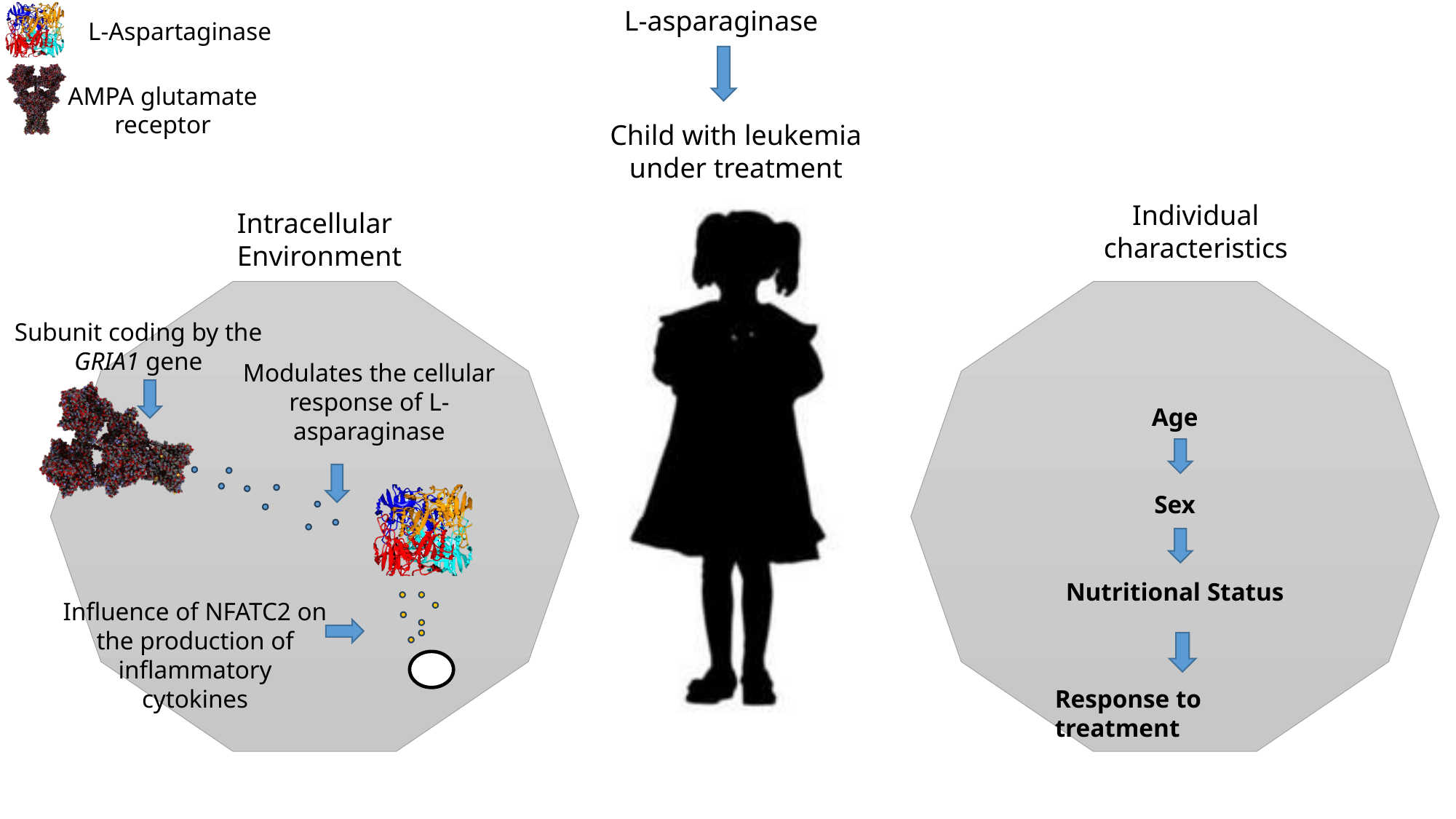

L-asparaginase
L-Aspartaginase
AMPA glutamate receptor
Child with leukemia under treatment
Individual characteristics
Intracellular Environment
Age
Sex
Nutritional Status
Subunit coding by the GRIA1 gene
Modulates the cellular response of L-asparaginase
Influence of NFATC2 on the production of inflammatory cytokines
Response to treatment
